# The Burden of Research on Trauma for Respondents: A Prospective and Comparative Study on Respondents Evaluations and Predictors

**DOI:** 10.1371/journal.pone.0077266

**Published:** 2013-10-21

**Authors:** Peter G. van der Velden, Mark W. G. Bosmans, Annette C. Scherpenzeel

**Affiliations:** 1 INTERVICT, Tilburg University, Tilburg, The Netherlands; 2 CentERdata, Tilburg University, Tilburg, The Netherlands; Maastricht University Medical Centre, The Netherlands

## Abstract

The possible burden of participating in trauma research is an important topic for Ethical Committees (EC's), Review Boards (RB's) and researchers. However, to what extent research on trauma is more burdensome than non-trauma research is unknown. Little is known about which factors explain respondents evaluations on the burden: to what extent are they trauma-related or dependent on other factors such as personality and how respondents evaluate research in general? Data of a large probability based multi-wave internet panel, with surveys on politics and values, personality and health in 2009 and 2011, and a survey on trauma in 2012 provided the unique opportunity to address these questions. Results among respondents confronted with these events in the past 2 years (N = 950) showed that questions on trauma were significantly and systematically evaluated as less pleasant (enjoyed less), more difficult, but also stimulated respondents to think about things more than almost all previous non-trauma surveys. Yet, the computed effect sizes indicated that the differences were (very) small and often meaningless. No differences were found between users and non-users of mental services, in contrast to posttraumatic stress symptoms. Evaluations of the burden of previous surveys in 2011 on politics and values, personality and health most strongly, systematically and independently predicted the burden of questions on trauma, and not posttraumatic stress symptoms, event-related coping self-efficacy and personality factors. For instance, multiple linear regression analyses showed that 30% of the variance of how (un)pleasant questions on trauma and life-events were evaluated, was explained by how (un)pleasant the 3 surveys in 2011 were evaluated, in contrast to posttraumatic stress symptoms (not significant) and coping self-efficacy (5%). Findings question why EC's, RB's and researchers should be more critical of the possible burden of trauma research than of the possible burden of other non-trauma research.

## Introduction

Whether participating in trauma and stressful life-events research is harmful for those exposed to these events or suffer from event-related mental health problems, has been the topic of (ongoing) concern and debate among researchers, mental health care professionals and funds for research. Similarly, Medical Ethical Testing committees (METC's) and Internal Review Boards will always evaluate new research proposals in this perspective [1], [2], [3], [4]. The background of the concerns is, among other ethical questions, that typical (posttraumatic) stress symptoms may prolong or exaggerate when respondents are confronted with questions that reminds them of the adverse event. Specific questions on for instance what has happened, what they heard, felt or did, or on the level of current event-related posttraumatic stress symptoms may elicit or intensify these stress symptoms. In fact, such symptoms are key symptoms of post-traumatic stress disorder: intense psychological distress at exposure to internal or external cues that symbolize or resemble an aspect of the traumatic event (such as questions in an interview or questionnaire), is one of the key symptoms of posttraumatic stress disorder (the B Criterion of PTSD: intrusive recollections) [5]. In this perspective it is conceivable that questions on trauma and life-events may be accompanied by (unpleasant) feelings and thoughts related to the event or its consequences and can, to a lesser or greater extent, cause distress and psychological burden: it is possible that affected respondents are vulnerable due to PTSD symptomatology and mental health problems.

However, what are the lessons of empirical research on this important topic? The systematic review of Newman and Kaloupek in 2004 [3] on risks and benefits of participating in research on trauma and life-events, identified 12 empirical studies on this issue. These studies included a large variety of persons confronted with potentially traumatic and life-events: disaster victims, trauma exposed refugees, military veterans, acute assault survivors, victims of partner violence, survivors of child abuse, psychiatric inpatients, college students and individuals who sought treatment after physical injuries due to stressful life events. Identified studies used very different timeframes, varying from day(s) to decades since the event (Vietnam veterans), and consisted of questionnaires and/or clinical interviews. The main conclusions of the reviewed studies were that the majority of study participants reported favorable cost-benefit appraisals regarding participation. A minority of participants did report experiencing strong emotions or more distress than anticipated as a result of the study, but for the most part even this group did not regret participation or evaluated the experience negatively, with most studies reporting no or only weak associations between study related distress and negative evaluations of study participation [5]. Participant characteristics associated with emotional reactions varied substantially among samples. These potentially risk factors included: posttraumatic stress symptoms, preexisting trauma related and unrelated distress, social vulnerability, younger or older age, multiple traumatic experiences, and physical injuries. Of course, these findings do not preclude the need for careful attention towards these ethical issues, since there does appear to be a (small) minority for whom the cost-benefit balance is not favorable [4], [5].

For example, Griffin and colleagues [6] examined this topic among female victims of domestic violence (N = 260; assessed in a 2-day period for 2–4 h./d., within 2 weeks- months post-assault) and acute physical/sexual assault (N = 170; assessed over a 2-day period for 3–5 h./.d within 3 weeks post-assault). Findings showed, among other things, that a small sub group evaluated the assessments as (very) distressing especially clinical interviews compared to paper-and-pencil and computer-based assessments. No significant differences were found between respondents with and without PTSD (neglecting the 1-month criterion). Almost all respondents (98%) reported that they either would be quite willing or might be willing to be assessed again in a similar way, despite the finding that 42% reported that they felt strong or very strong emotions during the assessment process.

Boscarino and colleagues [7] analyzed the data of a large study (N = 2368) after the 9/11 terrorists attacks in New York, and focused on residents (N = 1,173) who used mental health services (MHS) and residents who did not. Findings showed that the non-MHS users less often evaluated questions as stressful (12%) than the MHS users (28%). They also less often reported being emotionally upset at survey completion (1%) than the treatment sample (3%). With respect to overall rating of the surveys, no significant differences were found (generally negative 2% and 3% respectively). The study of Galea and colleagues [8], using the data of three cross-sectional population studies 1–2 months (N = 1,008), 4–5 months (N = 2,011), and 6–9 months (N = 2,775) after the terrorists attacks, showed that in total 1% of all respondents (N = 5,774) felt upset after the telephone interview and that 0.3% wanted assistance from a counselor.

Somewhat more recently, Resick and colleagues [9] assessed evaluations of the pre-treatment assessments among female survivors of childhood and/or adult interpersonal violence with PTSD (N = 100) who were seeking psychotherapy (3 days of assessment (3–4 h/d.). Findings showed that a small group evaluated the questionnaires as (very) distressing (8%) compared to the clinical interview (19%) and talking about the trauma (53%). Despite this distress, respondents generally indicated that they found the assessment procedure fairly interesting. Furthermore, the overwhelming majority of participants, (95%) was willing to participate in similar assessments in the future. This is striking given the fact that the assessment was an intensive and lengthy procedure. It is important to note that all participants met diagnostic criteria for PTSD before study participation, and levels of distress caused by the assessment procedure must be viewed in this light. Distress was not associated with the type of trauma, only with re-victimization. The authors also examined the independent associations between the evaluations and treatment outcomes and found that, interestingly, respondents who endorsed more burden were *more* likely to complete treatment.

Recently, Hebenstreit and DePrince [10] examined this topic in a 3-wave longitudinal study among women (N = 236) recently victimized by intimate partner abuse (IPA), i.e. the median time between arrest incident and recruitment was 26 days. An important feature of the recruitment procedure was that participants were only informed about the focus of the study on IPA when they came in for their first interview: initially, participants were told they would participate in a study about general health among women. In this way, the authors tried to assess the stressfulness of trauma-related questions among a relatively unbiased sample because participants were confronted with trauma related questions without much prior warning. If the trauma focus of a study had been advertised, it might have resulted in a biased sample of individuals who are more comfortable talking about their traumatic experiences. Outcomes showed that overall, across all three interviews, a positive benefit-to-cost ratio was indicated. For all three waves, scores on the positive factors were significantly greater than the neutral point, indicating agreement with statements about positive experiences and positive gains from the study. Similarly, scores on the negative factors were significantly less than the neutral points, indicating disagreement with statements regarding negative emotional reactions caused by the study. Multivariate analyses showed that evaluations as well as severity of IPA and PTSD symptomatology did not predict retention at the next interview.

Grubach and colleagues [11] examined the reactions to trauma research among a relatively small group of veterans (N = 51) suffering from severe mental illness and with multiple psychiatric hospitalizations (more than 2 months prior to study). The study was part of an open trial of an exposure based intervention for PTSD. A relatively large minority of the respondents (31%) evaluated the PTSD trauma research survey as distressing, while about two-thirds of the respondents was very satisfied (63%), evaluated the research as worthwhile (65%) and/or were likely to participate in similar future research (67%). Respondents who evaluated the research as very distressing were nonetheless willing to participate in the future. No differences in evaluations were found between respondents with and without PTSD.

In sum, empirical studies on this topic suggest that in most cases a variable minority of respondents evaluate the questions as stressful. Prevalence of stressfulness up to 31% among study samples was reported. Newman and Kaloupek [3] concluded “*Although a subset of participants report strong negative emotions or unanticipated distress, the majority of these do not regret or negatively evaluate the overall experience*”. The associations of the evaluations with PTSD symptomatology and other mental health problems differed across studies varying from no associations [11] to significant and medium associations [7]. However, as Newman and Kaloupek (2004) clearly noted, possible distress and harm may not be unique to trauma-related studies. In addition, some of the aforementioned studies, such as the study of Resick and colleagues [9] appear to be part of a treatment or intervention program, i.e. research questions were part of (long) intake and diagnostic process. The question remains to what extent findings among ‘clinical’ samples (where assessments are part of the intake and diagnosis process and inevitable) may be generalized to research among non-clinical samples [12] and vice versa.

Current studies on the perceived burden of research on trauma and life-events are -to the best of our knowledge- primarily focused on the evaluations of the particular (longitudinal) research the authors were conducting. Although Newman and Kaloupek [3] clearly noted that the possible burden of questions is not unique to trauma research, we are not aware of any study that systematically compared evaluations of trauma research with evaluations of the same participants on the burden of other research, i.e. evaluations on the burden of research conducted on other topics of (mental) health or on research completely outside this field. Such comparisons within the same study sample, or with comparable groups, are important because they provide insight in the relative burden of trauma research for the respondents: are they evaluated as more, less or equally difficult, clear, interesting, stimulating and (un)pleasant than studies that do not focus on potentially traumatic events and the personal consequences of these events?

Similarly, studies that focused on predictors of the burden were predominantly interested in event-related predictors such as the type or severity of the event and mental health disturbances such as PTSD (besides demographics). Johnson and Benight [2] however, focused on the associations between trauma-related coping self-efficacy among domestic violence survivors (N = 55) and evaluations of participating in trauma research, i.e. positive gain from participation, being more upset than anticipated, feelings of regret with regard to participating. Findings showed that coping self-efficacy was reversely related to being more upset than anticipated over and above PTSD symptomatology. To the best of our knowledge, there are no studies that assessed the associations between prospectively examined personality factors and evaluations of research on trauma and life-events. Including such variables may partially explain the self-reported burden of participating in research on trauma and life-events. For example, in a study among undergraduates Naemi and colleagues [13] found that intolerance of ambiguity and simplistic thinking interacted with the time spent on the survey predicted the participant's extreme responding: those who quickly completed surveys and were intolerant of ambiguity or were simplistic thinkers were most likely to exhibit extreme response styles (tendency to overuse the endpoints of Likert-type scales). It is completely unclear to what extent the perceived burden of trauma research can be attributed to a possible general tendency of respondents to experience questions as burdensome regardless of the topic of the research. In the latter case, evaluations on the burden of previous non-trauma and non-life-events research would be predictive of evaluations on the burden of trauma and life-events research.

### Research questions

Aim of the present longitudinal comparative study is to gain more insight in the relative burden of participating in trauma research and predictors of the burden to fill this gap of scientific and practically relevant information. More specifically, main research questions of the present study are:

1. To what extent do respondents evaluate questions on trauma as more, less or equally burdensome than questions in surveys on other topics such as on a.) health, b.) personality, and c.) politics and values? With burdensome we mean that the questions were difficult to answer, were not sufficiently clear for respondents, got respondents thinking about things, were not interesting for them, and that respondents did not enjoy answering the questions.
2. To what extent do current posttraumatic stress symptoms, mental health services utilization, demographics, prospectively assessed personality factors and earlier evaluations on the burden of research on other topics such as health, personality and politics and values, predict the burden of questions on trauma?

## Materials and Methods

### Participants: The LISS panel

The data for this study were collected in the LISS panel (Longitudinal Internet Studies for the Social Sciences). This panel is the principal component of the MESS project, operated by the CentERdata research institute in Tilburg, the Netherlands. It consists of almost 8000 individuals that complete online questionnaires every month. The LISS panel is based on a traditional random sample drawn from the population register by Statistics Netherlands. Persons not included in the original sample cannot participate, so there can be no self-selection. People without a computer or Internet connection are provided with equipment to participate. LISS panel members complete online questionnaires every month, for which they get an incentive of €15, – per hour of interview time. The household attrition rate is on average 10% per year. Thus, respondents are periodically invited to participate in internet surveys on distinct topics with an estimated duration of 30–45 minutes for each survey.

### The surveys

For the present study we extracted data from 6 surveys conducted in 2009 and 2011 on personality, health and politics and values (all three in 2009 and all three in 2011). In May 2012, the topic of our survey was on trauma, i.e. potentially traumatic events, event-related coping self-efficacy and posttraumatic stress symptoms. All 7 surveys ended with five identical questions on the burden of the survey. For the present study we focused on participants who, according to the last survey on trauma, were confronted with one or more potentially traumatic events in the past 2 years before the survey on trauma (backward selection). The overall monthly response of the 7 surveys varied between 67% and 78% (for details about the 7 surveys see appendix S1).

### Board review

Proposals for studies, such as the present study on trauma, in the LISS panel are evaluated by the Board of Overseers of the MESS project (Internal Review Board). The Board consists of 10 prominent scientists from 8 different Dutch universities and Statistics Netherlands. One member is assigned to write a short review using five criteria: scientific relevance, suitability for the LISS panel, feasibility, target group, and length. These criteria are further elaborated on the website www.lissdata.nl. On the basis of one or more referee report(s), the Board of Overseers will take one of three decisions: accept, revise and resubmit, or reject. Occasionally, the Board recommends a pretest before implementing a questionnaire or experiment in the LISS panel itself, for example for survey questions on sensitive topics, economic experiments that might be difficult to understand for socioeconomic groups with low financial literacy, or high-frequency measurement of weight and other biomarkers. Such pretests help to decide whether the questions are feasible at all, do not reduce future participation, and can be useful in choosing between various wordings of the questions or in shaping the design of an experiment. The board approved our proposal for the study on trauma.

### Informed consent and ethical approval

During the recruitment of the panel, respondents who agreed to participate in the panel received a confirmation e-mail, and a letter with login code. With the login code provided they could confirm their willingness to participate and immediately start the first interview. This confirmation procedure, following the consent to participate given to the interviewer, ensured the double consent of each respondent to become a panel member and participate in the monthly panel questionnaires. The present study was part of the normal monthly panel questionnaires, for which no specific consents were asked after the general consent for panel participation was given.

It has to be noted that Medical Ethics Committee approvals for questionnaire research among adults are only required in the Netherlands when the (expected) burden for all respondents is (very) great, such as (very) large and time consuming questionnaires (in contrast to research among children when approval is needed), and RCT-studies where respondents have to or must follow certain protocols or instructions (see www.ccmo-online.nl). In case of doubt, METC's can help to examine if the proposed research project needs a formal METC approval. Similar research with semi-structured interviews and questionnaires among victims of a disaster in the past was evaluated by an METC (METC UMC Utrecht University, the Netherlands) as not requiring a METC approval. For these reasons we did not ask for a formal METC approval.

### Measures

For the present study, the following variables were extracted. Besides gender, age, education level, and personal income (after Tax and in euros) that were (re-) assessed for the last time in our survey on trauma (2012), data was used on evaluations on the burden of each survey, on potentially traumatic events, posttraumatic stress reactions (PSS), coping self-efficacy and on personality factors. The complete questionnaires of the earlier surveys are available online (see www.lissdata.nl/dataarchive/study_units/view/1), as will the survey on trauma 2012 at a later stage.

#### Burden of questions

To examine how burdensome the questions of each of the surveys were, the 7 surveys ended with the following 5 questions: 1.) Was it difficult to answer the questions?, 2.) Were the questions sufficiently clear?, 3.) Did the questionnaire get you thinking about things?, 4.) Was it an interesting subject?, and 5.) Did you enjoy answering the questions? Items were scored on 5-point likert scales ranging from definitely no (1) to definitely yes (5).

#### Potentially traumatic events

To assess potentially traumatic events (PTE) in the past two years, respondents were administered a trauma and life-events exposure list of 10 potentially traumatic events (serious threat, physical violence, robbery, traffic accident, airplane accident, fire, burglary, serious infection such as HIV, legionella and poison, sexual violence or abuse, death of a significant other or colleague).These experiences were derived from a life-events and trauma list [14]. We added an open question with regard to having experienced other potentially traumatic events not listed. Reported potentially traumatic events to this open question were severe illness (self or other such as cancer), being confronted with another (severe) type of accident, significant other was confronted with (severe) accident, and theft. A very small number of respondents reported being confronted with miscarriage or with suicide. For the present study, these respondents were assigned to the sub group reporting being confronted with the death of a significant other.

In case respondents had been confronted with more than one event in the two year period, they were asked to focus on the most recent and stressful event. In case a respondent had no experiences with potentially traumatic events, he or she could skip a very large proportion of the survey. In the present study we focus on potentially traumatic events, thus excluding stressful life-events such as divorce, loss contact with children, and severe problems with partner or colleagues (responses to open question). In addition, respondents were asked when the event happened in the past two years (We also asked additional questions about the characteristics of the event such as recalled stress during event, sustained personal injuries and needed medical care, but these data were not used for the present study). For the present study we formed two categories of potentially traumatic events: events excluding the death of a significant other or colleague (1) and events including the death of a significant other (2). With respect to time between the event and survey, we made a distinction between 0–1 year (1) and 1–2 years (2) before the survey on trauma.

#### Posttraumatic stress symptoms

Current event-related posttraumatic stress reactions (PSS) were assessed in the survey on trauma 2012, using the 15-item Impact of Event Scale [15], [16] covering event-related intrusions and avoidance reactions. Additionally, seven items of the 22-item IES-R covering event-related hyper arousal were added [17], [18], [19] to the IES-15 (using the likert-scales of the IES 15-item version; 1 = not at all, 4 = often). We will call this expanded version of the original IES the IESplus. The trauma exposure list and IESplus were only administered in the survey on trauma (2012).

#### Personality factors

Personality factors were examined using the IPIP [20], [21], [22], [23]. This is a personality measure based on the Big-five factor structure of personality which distinguishes 5 major dimensions of personality: neuroticism, extraversion, openness to experience, agreeableness and conscientiousness. The 50-item version was used, in which all five personality factors are assessed by 10 items (items scored on a 5 point scale, ranging from 1 very inaccurate, to 5 very accurate). The IPIP has been proven to have a consistent factor structure [24]. For our analyses we used the most recent personality survey in the LISS-panel (2011).

#### Coping self-efficacy

A brief 7-item version [ref] of the 20 item Coping Self-Efficacy (CSE) Scale [25], [26] was used to assess event-related CSE. For each item, respondents rated their perceived efficacy on dealing with different consequences of the potentially traumatic event they experienced on a 7 point scale (e.g. ‘resuming normal life’; ‘dealing with frightening images or dreams about the event’; ‘being optimistic since the event’). The scores range from 1 (‘I am completely incapable of’) to 7 (‘I am perfectly capable of’).

#### Mental health services

Respondents were asked whether they ever used mental health services (psychologist, psychiatrist or psychotherapist) in the past and/or in the past 12 months.

### Statistical analyses

We first selected respondents from the survey on trauma 2012 who were exposed to potentially traumatic events (N = 2348) in the past 2 years. In total, 950 of 2137 respondents participated in all 7 surveys and were confronted with potentially traumatic events (see Table 1). We focus on the latter group of 950 respondents because it enabled perfect comparisons of their evaluations on the survey on trauma and their evaluations of other surveys, i.e. within-group comparisons using paired t-tests (we did not conduct mutual comparisons of previous evaluations). Estimates of effect sizes of significant differences between evaluations were computed using Cohen's d for dependent samples (Cohen's D = *t*/√N). Control analyses were conducted using the data of all other respondents of the two surveys that were compared (resulting in different numbers).

**Table 1:** Demographic characteristics respondents (N = 950).

	N	%
Gender		
males	451	47.5
females	499	52.5
Education		
primary school	69	7.3
junior high school	258	27.2
senior high school	106	11.2
junior college	223	23.5
college	226	23.8
university	66	6.9
unknown (missing)	2	.2
Income		
0 No income	73	7.7
EUR 500 or less	43	4.5
EUR 501 to EUR 1000	182	19.2
EUR 1001 to EUR 1500	177	18.6
EUR 1501 to EUR 2000	215	22.6
EUR 2001 to EUR 2500	102	10.7
EUR 2501 to EUR 3000	53	5.6
EUR 3001 to EUR 3500	31	3.3
EUR 3501 to EUR 4000	8	.8
EUR 4001 to EUR 4500	3	.3
EUR 4501 to EUR 5000	2	.2
EUR 5001 to EUR 7500	6	.6
More than EUR 7500	3	.3
I really dont know (missing)	9	.9
I prefer not to say (missing)	39	4.1
Unknown (missing)	4	.4
Event		
Accident, (sexual) violence, fire, disaster, illness, etc.	337	35.5
Death significant other or colleague	613	64.5
Time event		
0–1 year ago	581	61,2
1–2 years ago	369	38,8
Age (rage 19–92 year)	M	SD
in years	54.43	15.11

In addition, series of hierarchical multiple linear regression analyses (abbreviated as MR-analyses) were used for the second research question, i.e. to examine the independent predictive values of current posttraumatic stress symptoms (step 1), event-related coping self-efficacy (step 2) demographics (step 3: age, gender, education, income), five personality factors prospectively assessed in 2011 (step 4: extraversion, agreeableness, conscientiousness, neuroticism, openness), and evaluations of the surveys on health, personality and politics and values conducted in 2011 (step 5: these items were paired, i.e. for predicting (lack of) pleasure with regard to the survey on trauma, the same items with regard to the health, personality and politics surveys were entered at step 5), and time and type of the event (step 6).

Dependent variables were the answers of respondents on the 5 questions evaluating the burden of the trauma research. Missing values across survey were almost absent; only for net income several respondents refused to provide information. For this reason we choose to use the SPSS option pair-wise deletion instead of list-wise deletion in the MR-analyses. To rule out multicollinearity we first assessed inter-correlations as well as variance inflation factors (*VIF's*). Findings showed no indications for multicollinearity, i.e. all correlations were ≤.65 and all VIF's in full models were ≤2.15.

Finally, we examined to what extent respondents who used mental health services (MHS) in the past (ever or in past year) differed in their evaluations from respondents who never used MHS using ANOVA's, while controlling for demographics. IBM SPSS statistics 20 was used to perform all analyses.

## Results

### Characteristics respondents

Demographic characteristics of 950 respondents are presented in Table 1.

### Comparisons of evaluations of the burden of questions on trauma

Table 2 shows that, compared to all six previous surveys, the questions of our survey on trauma were evaluated significantly and systematically as less pleasant, although the mean score (M = 3.50) was above 3 (neutral), indicating a positive average evaluation. According to the Cohen's d criteria, the differences between the evaluations may be considered (very) small (Cohen's D<.5). Meanwhile, respondents evaluated the survey on trauma as somewhat more stimulating to think about things than all other surveys. However, the difference between our survey on trauma and the survey on health 2009 was much larger (d = −.65). Furthermore, the questions on trauma were evaluated as significantly more difficult to answer than the questions of other surveys, although all effects sizes may be considered (very) small. Finally, table 2 shows that the questions on trauma were not systematically evaluated as more or less a.) sufficiently clear and b.) as more interesting than the other surveys.

**Table 2:** Pair-wise comparisons of burden research on politics and values, personality and health, with burden of research on trauma (N = 950).

		Evaluations burden of research																									
		Enjoy answering						Questionnaire get					Interesting					Difficult to answer					Questions				
		questions						thinking about things					subject					the questions					sufficiently clear				
	N					Cohen						Cohen					Cohen					Cohen					Cohen
Research topic	Total	M	SD	t	p	D	M		SD	t	p	D	M	SD	t	p	D	M	SD	t	p	D	M	SD	t	p	D
Politics&values 2009	950	**3.77**	**0.94**	**7.03**	**<.001**	**0.23**	2.99		1.09	−10.28	<.001	−0.33	**3.74**	**0.96**	**0.11**	**ns.**		2.17	1.16	−2.52	.01	−0.08	**4.08**	**1.06**	**−2.77**	**.01**	**−0.09**
Politics&values 2011	950	**3.87**	**0.90**	**11.59**	**<.001**	**0.38**	3.19		1.13	−6.27	<.001	−0.20	**3.81**	**0.91**	**2.05**	**.04**	**0.07**	2.16	1.26	−2.94	.00	−0.10	**4.21**	**0.85**	**0.32**	**ns.**	
Health 2009	950	**3.73**	**0.97**	**6.51**	**<.001**	**0.21**	2.63		1.10	−19.91	<.001	−0.65	**3.53**	**0.95**	**−5.50**	**.00**	**−0.18**	1.73	0.99	−11.59	<.001	−0.38	**4.23**	**1.00**	**0.79**	**ns.**	
Health 2011	950	**3.85**	**0.97**	**10.78**	**<.001**	**0.35**	3.00		1.18	−11.56	<.001	−0.37	**3.77**	**0.91**	**1.17**	**ns.**		1.71	1.01	−12.57	<.001	−0.41	**4.37**	**0.82**	**5.40**	**<.001**	**0.18**
Personality 2009	950	**3.79**	**0.95**	**7.99**	**<.001**	**0.26**	3.30		1.11	−2.80	<.001	−0.09	**3.84**	**0.94**	**2.73**	**.01**	**0.09**	2.92	1.29	12.38	<.001	0.40	**3.94**	**0.95**	**−6.90**	**<.001**	**−0.22**
Personality 2011	950	**3.81**	**0.98**	**8.95**	**<.001**	**0.29**	3.37		1.11	−0.88	ns.		**3.83**	**0.95**	**2.88**	**.00**	**0.09**	2.31	1.31	0.13	ns.		**4.12**	**0.91**	**−2.24**	**.03**	**−0.07**
Trauma 2012	950	**3.50**	**1.09**				3.41		1.21				**3.74**	**1.04**				2.30	1.40				**4.20**	**0.99**			

p is p-value paired t-test, 2-tailed.

ns. = Not significant.

These outcomes relate to affected respondents who participated in all surveys (N = 950). This may skew the study sample in favor of respondents who are more comfortable answering questions in surveys with different focus. We therefore repeated all pair-wise comparisons among affected participants who did not participate in all surveys (resulting in different sizes for each comparison). Outcomes of this control analyses hardly differed from the outcomes presented in table 2 and showed the same pattern (see appendix S2).

### Predicting evaluations on the burden of questions on trauma

We first conducted MR-analyses with the evaluation of the surveys in 2012 among the group of 950 respondents. The summary statistics of this analyses, i.e. R^2^, Δ R, F^change^ and p- values of each step, are presented in table 3. Posttraumatic stress symptoms (PSS) at step 1, did not significantly explain the variance of (not) enjoying the questions and (not) finding the questions sufficiently clear and did not strongly predict the other evaluations (.01%<R^2^ <.06). The same pattern can be observed with respect to coping self-efficacy, demographics and personality factors in step 2, 3 and 4 respectively. PSS and coping self-efficacy at step 2, accounted for between about 4–8% of the variance of the evaluations. Results show that the evaluations of previous surveys together contributed much more to the total explained variance of the evaluations of the questions on trauma, than any of the other predictors in table 3: they accounted for about 18–38% of the variance of the evaluations on the burden of the trauma research over and above all other predictors. Time elapsed since event and type of event were not significant predictors in our models.

**Table 3:** Explained variance of each step in multiple regression analyses predicting burden of research on trauma (N^max^ = 950).

	Enjoy answering				Questionnaire get thinking				Interesting				Difficult to answer				Questions sufficiently			
	questions				about things				Subject				the questions				clear			
Group (number predictors each step)	Adj. R^2^	R^2ch^	F^change^	p	Adj. R^2^	R^2ch^	F^change^	p	Adj. R^2^	R^2ch^	F^change^	p	Adj. R^2^	R^2ch^	F^change^	p	Adj. R^2^	R^2ch^	F^change^	p
**Step 1**																				
Posttraumatic stress symptoms (1)	**0.00**	**0.00**	**1.3**	**ns.**	0.06	0.06	55.7	<.001	**0.02**	**0.03**	**23.2**	**<.001**	0.05	0.05	45.3	<.001	**0.00**	**0.00**	**0.0**	**ns.**
**Step 2**																				
Coping self-efficacy (1)	**0.05**	**0.05**	**44.5**	**<.001**	0.06	0.00	0.8	ns.	**0.04**	**0.02**	**15.3**	**<.001**	0.08	0.03	29.6	<.001	**0.04**	**0.04**	**40.3**	**<.001**
**Step 3**																				
Demographics (4)	**0.07**	**0.03**	**7.1**	**<.001**	0.06	0.01	2.1	ns.	**0.05**	**0.01**	**3.3**	**.01**	0.08	0.00	0.9	ns.	**0.05**	**0.01**	**2.1**	**ns.**
**Step 4**																				
Personality factors (5)	**0.09**	**0.02**	**4.3**	**<.001**	0.09	0.04	7.2	<.001	**0.09**	**0.04**	**8.0**	**<.001**	0.09	0.02	4.6	<.001	**0.09**	**0.05**	**9.4**	**<.001**
**Step 5**																				
Evaluations surveys in 2011 (3)	**0.38**	**0.29**	**138.5**	**<.001**	0.48	0.38	221.3	<.001	**0.34**	**0.25**	**114.0**	**<.001**	0.28	0.19	77.9	<.001	**0.27**	**0.18**	**73.4**	**<.001**
**Step 6**																				
Event (2)	**0.38**	**0.00**	**0.26**	**ns.**	0.48	0.00	0.09	ns.	**0.34**	**0.00**	**0.69**	**ns**	0.28	0.00	0.02	ns.	**0.28**	**0.00**	**0.14**	**ns.**

Adj. R^2^  = Adjusted R^2^.

R^2ch^  = R^2^ change.

ns. = not significant.

Table 4 presents detailed information on the outcomes of the MR-analyses, i.e. the beta's (B), standard errors of B, standardized Beta (β), and p-values of each predictor at step 6 (full model, outcomes of the first 5 steps are available on request).

**Table 4:** Full models multiple regression analyses predicting burden of research on trauma (Step 6, N^max^ = 950).

	Enjoy answering				Questionnaire get				Interesting				Difficult to answer				Questions			
	questions				thinking about things				subject				the questions				sufficiently clear			
	B	SE	β	p	B	SE	β	p	B	SE	β	p	B	SE	Β	p	B	SE	β	p
PSS	**0.00**	**0.00**	**0.00**	**ns.**	0.01	0.00	0.16	<.001	**0.01**	**0.00**	**0.13**	**<.001**	0.01	0.00	0.14	<.001	**0.00**	**0.00**	**0.07**	**ns.**
CSE	**0.02**	**0.00**	**0.17**	**<.001**	−0.01	0.00	−0.03	ns.	**0.01**	**0.00**	**0.06**	**ns.**	−0.02	0.01	−0.13	<.001	**0.02**	**0.01**	**0.16**	**.001**
Education	**−0.04**	**0.02**	**−0.06**	**ns.**	0.00	0.02	−0.01	ns.	**−0.02**	**0.02**	**−0.03**	**ns.**	−0.01	0.03	−0.01	ns.	**−0.04**	**0.02**	**−0.06**	**ns.**
Age	**0.00**	**0.00**	**−0.02**	**ns.**	0.00	0.00	−0.04	ns.	**0.00**	**0.00**	**0.00**	**ns.**	0.01	0.00	0.06	ns.	**0.00**	**0.00**	**−0.02**	**.045**
Income	**0.00**	**0.02**	**0.00**	**ns.**	−0.02	0.02	−0.03	ns.	**−0.01**	**0.02**	**−0.01**	**ns.**	0.00	0.03	−0.01	ns.	**−0.01**	**0.02**	**−0.03**	**ns.**
Gender	**−0.02**	**0.07**	**−0.01**	**ns.**	−0.13	0.07	−0.05	ns.	**0.00**	**0.07**	**0.00**	**ns.**	−0.14	0.10	−0.05	ns.	**0.05**	**0.07**	**0.02**	**ns.**
Extraversion	**0.00**	**0.01**	**−0.02**	**ns.**	−0.01	0.01	−0.05	ns.	**0.00**	**0.01**	**−0.02**	**ns.**	0.00	0.01	0.00	ns.	**0.00**	**0.01**	**−0.02**	**ns.**
Agreeableness	**0.00**	**0.01**	**0.01**	**ns.**	0.02	0.01	0.08	.005	**0.02**	**0.01**	**0.07**	**.023**	0.01	0.01	0.04	ns.	**0.02**	**0.01**	**0.12**	**.001**
Conscientiousness	**−0.01**	**0.01**	**−0.03**	**ns.**	0.01	0.01	0.03	ns.	**−0.01**	**0.01**	**−0.04**	**ns.**	0.00	0.01	0.01	ns.	**0.01**	**0.01**	**0.07**	**ns.**
Neuroticism	**0.01**	**0.00**	**0.05**	**ns.**	0.00	0.00	−0.01	ns.	**0.00**	**0.00**	**0.00**	**ns.**	−0.01	0.01	−0.05	ns.	**0.01**	**0.01**	**0.04**	**ns.**
Openness	**0.00**	**0.01**	**0.00**	**ns.**	0.00	0.01	0.01	ns.	**0.01**	**0.01**	**0.05**	**ns.**	−0.01	0.01	−0.04	ns.	**0.01**	**0.01**	**0.03**	**ns.**
Politics 2011	**0.26**	**0.05**	**0.22**	**<.001**	0.29	0.04	0.27	<.001	**0.17**	**0.04**	**0.15**	**<.001**	0.24	0.04	0.22	<.001	**0.00**	**0.03**	**0.00**	**ns.**
Health 2011	**0.33**	**0.04**	**0.29**	**<.001**	0.26	0.03	0.26	<.001	**0.35**	**0.04**	**0.31**	**<.001**	0.24	0.04	0.17	<.001	**0.16**	**0.03**	**0.16**	**<.001**
Personality 2011	**0.18**	**0.04**	**0.16**	**<.001**	0.24	0.03	0.22	<.001	**0.20**	**0.04**	**0.18**	**<.001**	0.21	0.04	0.19	<.001	**0.18**	**0.03**	**0.18**	**<.001**
Type event	**−0.04**	**0.06**	**−0.02**	**ns.**	0.01	0.06	0.01	ns.	**−0.07**	**0.06**	**−0.03**	**ns.**	0.00	0.08	0.00	ns.	**0.00**	**0.06**	**0.00**	**ns.**
Time	**−0.02**	**0.06**	**−0.01**	**ns.**	−0.02	0.06	−0.01	ns.	**−0.02**	**0.06**	**−0.01**	**ns.**	0.02	0.08	0.01	ns.	**−0.02**	**0.02**	**−0.03**	**ns.**

PSS  = posttraumatic stress symptoms.

CSE = coping self-efficacy.

ns. = not significant.

The five personality factors neuroticism, extraversion, conscientiousness and openness did not significantly explain variance of the five questions on the burden of trauma research over and above all other variables in table 3. Agreeableness was independently and positively predictive of ‘get thinking about things’ due to the questions, evaluating the questions as interesting, and of evaluating the questions as sufficiently clear although the β's were much lower than the β's of previous evaluations. Posttraumatic stress symptoms (PSS) were independently and positively, predictive of ‘get thinking about things’, evaluating the questions as ‘interesting topic’ but also of evaluating the questions as ‘difficult to answer’, although less than previous evaluations. PSS were not significantly associated with evaluating the questions as more or less pleasant or sufficiently clear in the full models. Results show that coping self-efficacy was independently and positively associated with evaluating the questions of our trauma research as pleasant, the questions on trauma as interesting and sufficiently clear, and was negative associated with evaluating the questions as difficult to answer. Findings show that type and time of event were not associated with evaluations.

Because of these findings, we repeated the 5 MR-analyses using the data of evaluations of the surveys on politics and values, health and personality conducted in 2009 instead of the evaluations of the surveys in 2011. Findings showed that evaluations of surveys 3 years earlier again were significant and independent predictors of the perceived burden of the questions on trauma although less powerful, especially for evaluating the questions as clear (R^2 change^ were.19,.24,.14,.13,.06 at step 5 for (lack of) enjoy, get thinking, interesting subject, difficult questions and clear question respectively: for further details see appendix S3).

Table 5 contains de outcomes of the ANOVA's on the evaluations among respondents who never used mental health services (MHS), respondents who ever used MHS, and respondents who used MHS in the past year. Findings show that these sub groups do not differ in evaluations on the burden of our study on trauma, although they differed significantly in posttraumatic stress symptoms (M^never used MHS^ = 18.74, SD = 17.49; M^ever used MHS^ = 22.50, SD = 21.31; M^use MHS past year^ = 27.88, SD = 24.24 respectively, F(2,895) = 11.92, p<0.001).

**Table 5:** Differences in burden of research on trauma between non-MHS users, ever MHS users and MHS users in the past year.

	Use of mental health services (MHS)							
	Never (N = 652)		Ever (N = 163)		Past year (N = 81)			
Evaluations survey on trauma	M	SD	M	SD	M	SD	F(2,895)^1^	p-value
difficult to answer the questions	**2.30**	**1.42**	2.26	1.35	**2.41**	**1.41**	.477	ns.
questions sufficiently clear	**4.16**	**1.03**	4.35	0.85	**4.16**	**0.93**	2.391	ns.
questionnaire get thinking about things	**3.36**	**1.23**	3.48	1.14	**3.62**	**1.14**	1.600	ns.
interesting subject	**3.73**	**1.06**	3.78	0.93	**3.83**	**1.03**	.613	ns.
enjoy answering questions	**3.56**	**1.08**	3.42	1.07	**3.28**	**1.22**	1.940	ns.

1 F -value of main effect (ANOVA) of mental health services utilization with age, gender, net income and education as covariates (covariates entered first). The total number is smaller due to missing values covariates (see also table 1).

ns. = not significant.

## Discussion

### Predictors of burden

Our unique longitudinal and comparative study adds new insights in how the possible burden of trauma research can be understood and explained. While previous research and debates on this issue predominantly focused on the associations between trauma, PTSD symptomatology and experienced burden, besides demographics, our study clearly shows that previous evaluations of studies in 2011 that were not focused on trauma at all were, without any doubt, the most powerful and independent predictors over and above posttraumatic stress symptoms, coping-self-efficacy, demographics and personality factors. Interestingly, evaluations of the burden of surveys 3 years earlier were still independent predictors of current evaluations of our research on trauma.

Nevertheless, our findings are in line with the outcomes of studies on the predictive power of response behavior. Two studies [27], [28] using data of the LISS panel [29] have shown that attrition and inactive periods were much more related to respondents' past response behavior than to demographic characteristics: Skipping a questionnaire or completing questionnaires irregularly turned out to be the best predictors for future drop-out.

Negative evaluations of questions in trauma research were explained by PTSD symptomatology, demographics and prospectively examined personality factors to a (very) limited extent. Importantly, these findings cannot be attributed to recall bias since these evaluations were given at the time of the particular survey in the past. Nevertheless, the directions of the significant independent associations between PTSD symptomatology and coping self-efficacy were in line with previous research: more symptoms and less coping self-efficacy was associated with (some) more reported burden [2], [3], [4].

The variable ‘complete participation’ was not an independent predictor over and above all other study variables. This finding clearly suggests that, although future research is warranted, our findings were not biased by the fact that respondents participated in several surveys. In other words, our results do not disregard that respondents may evaluate questions on trauma as burdensome -one of the topics that METC's and IRB's will always look at- but are solid enough to fundamentally question the one-dimensional focus on posttraumatic stress symptoms as an indicator or marker of the burden of trauma research. Moreover, our findings in combination with the outcomes of previous research [1], [2], [3], [4], [6], [9], [10], [11], [12] indicate that the current almost one-dimensional focus on the presumed burden of research on trauma among adults, i.e. vulnerability due to PTSD symptomatology, of METC's and IRB's needs a revision. Results show that, when society and science ask for research on trauma to improve our knowledge and increase the effectiveness of possible interventions in one way or another, there is very little reason for METC's and IRB's to be more critical with respect to the burden of trauma research than they should to the possible burden of other non-trauma related research.

Of course, our study is ‘limited’ to questionnaires administered via internet and therefore findings may not be applicable to the relative burden of clinical interviews. Results of previous studies suggest that clinical interviews may be more burdensome than questionnaires. However, since many questions in questionnaires and interviews are similar or almost similar in wording, it remains to be clarified why personal interviews, as inevitable part a of the intake and diagnosis process, may be more burdensome. Why does the presence of an interviewer evoke more ‘negative’ evaluations in some cases? The study of Dill and her colleagues has shown that, with respect to physical or sexual abuse, that questionnaires are not accompanied by lower disclosure rates than interviews [30].

### Differences with other surveys

The LISS panel data created an unique opportunity to examine to what extent research on trauma and life-event is evaluated as more burdensome than non-trauma research on politics and values, mental health and personality among a large sample of respondents confronted with potentially traumatic events and life-events (besides the opportunity to prospectively examine the predictive values of personality and previous evaluations). Although previous studies did not examine these differences, our results appear to be in line with these studies showing that questions in research on trauma and life-events were systematically evaluated as less pleasant than previous non-trauma and life-events studies. However, computed effect sizes indicated that the differences were limited and, with regard to the other 4 evaluative questions, in many cases meaningless. Control analyses among those who did not participate in all surveys showed the same patterns. These finding are relevant and again show that questions on trauma may be burdensome, but must be interpreted with the outcomes on predictors of the evaluation on the burden of trauma research strongly in mind. In addition, our findings on differences between mental health services users and non-users differed with the findings of Boscarino and colleagues [7].

### Strengths and limitations

Affected respondents in our study were drawn from a large probability sample of Dutch adult residents. The response rates of the 7 surveys were very acceptable (66%< Response <81%) and evaluations of previous surveys were not based on retrospectively collected data that is (very) sensitive to recall bias. We are not aware of any study on the burden of trauma research, using our comparative and longitudinal approach.

As said, affected respondents who participated in all surveys may have skewed the study sample in favor of respondents who were more comfortable answering questions in surveys with different focus. Respondents received a small financial incentive, which may have reduced possible critics. Nevertheless, re-analyses using the data of all respondents who participated in the survey on trauma and in each other survey in 2011 and with ‘complete participation’ as an additional predictor did not affect our findings. Similarly, analyses among respondents who did not participate in all surveys showed the same pattern of differences in evaluations as those who participated in all surveys. These findings clearly suggests that our results are not, or to a very limited extent, influenced by the specific characteristics of our study samples, i.e. that they voluntarily participated in an internet panel with multiple surveys. On the other hand, it must be noted too that we do not know how often respondents in other studies participated in previous scientific or applied research projects. To the best of our knowledge, none of the previous studies on the burden of trauma research explicitly assessed this topic and therefore we cannot rule out the possibility that a similar self-selection process took place to a lesser or greater extent. The three-wave study Hebenstreit and DePrince [10] found that evaluations did not predict retention at the next interview but the self-selection may have taken place before. Nevertheless, future research is warranted since our study is the first prospectively examining this issue.

We used 5 identical questions across all surveys to examine the burden of the questions in each survey, and the one on trauma in particular. We did not specifically ask how upset respondents felt (or if participation was more stressful than expected beforehand) during the survey as in the Reactions to Research Participation Questionnaire (RRPQ) [2], but asked a similar question ‘Did you enjoy answering the questions’ with the answer categories ‘definitely no’ to ‘definitely yes’. It is possible in principle that questions on feeling tense for instance would be predicted by posttraumatic stress symptoms and coping-self-efficacy to a larger extent, but we believe that is not very likely given the pattern of our findings.

We did not ask respondents whether, due to the nature of questions, they needed professional help or sought help. Of course, seeking help is not a negative consequence per se: questions may help respondents to clarify or realize that seeking professional help is perhaps a necessary step given the problems they already encountered before participating in the survey. Moreover, online screening instruments -aimed at stimulating victims to seek help when intense posttraumatic stress symptoms do not appear to decrease- are becoming more and more popular. Insight in possible positive effects is as important as insight in possible negative effects since it may provide a more balanced picture of the consequences of participating in trauma research [3], [4].

In addition, like other studies on the burden of trauma research this study focused on adults. It is unknown whether our findings, and those of others, are applicable to children and young adolescents. Compared to research among adults, there is an huge gap of information with regard to these groups. Therefore, research on this topic is warranted, also because Medical Ethical Testing Committees or Internal Review Boards are attentive and sensitive for possible negative effects of participating in trauma research for these groups. In the Netherlands for instance, ethical rules prescribe informed consents of parents when conducting research among 12–16 years children and young adolescents, besides of course informed consent of the young participants, when studies are being conducted that fall under the law WMO (Law Medical Research, see www.ccmo.nl). To the best of our knowledge, it is unknown whether children and young adolescents are more, equal or less vulnerable than adults when participating in trauma research.

### Future research

Aim of the present study was to examine whether trauma research was evaluated as more, equal or less burdensome than non-trauma research, and to identify predictors of evaluations on the burden of trauma research. Surprisingly, evaluations of previous non-trauma research were systematically strong independent predictors of evaluations of trauma research in contrast to especially current posttraumatic stress reactions. In other words: findings indicate that in order to better understand evaluations of the burden of trauma research, we predominantly have to look at how participation in previous research was evaluated, presumably regardless of the topic of the research. Coping self-efficacy, PTSD symptomology, demographics (age) and personality factors (agreeableness) did, in various combinations, explain 8–13% of the variance of the reported burden of trauma research in 2012 compared to 18–39% by three previous evaluations of surveys in conducted in 2011. These findings raises the question if there is some kind of ‘burden-proneness’-response style resulting in a trajectory of relative ‘negative’ evaluations across surveys. It was outside the aim of the present study to address this question -in fact it is an outcome of the present study- but future research on this topic is warranted.

## Supporting Information

Appendix S1 (DOCX)

Appendix S2 (DOCX)

Appendix S3 (DOCX)
