# Supplementary material for: The Burden of Research on Trauma for Respondents: A Prospective and Comparative Study on Respondents Evaluations and Predictors
Source: PLoS One. 2013 Oct 21;8(10):e77266. doi: 10.1371/journal.pone.0077266 (PMC3804544; doi:10.1371/journal.pone.0077266)
Supplement: Appendix S1 — (DOCX) [file pone.0077266.s001.docx]

Appendix S1. Overview samples and respondents.

|  | **Number** | **Sampling period** |  |  |  | **Confronted with PTE^1^** | |
| --- | --- | --- | --- | --- | --- | --- | --- |
| **Topic survey** | **Items** | **Invite respondents** | **Re-invite non-responders** | **Response** | **N total** | **N^2^** | **N listwise^3^** |
| 1 Politics and values 2009 | 159 | 1-12-2008 to 31-12-2008 | 5-1-2009 to 28-1-2009 | 72.80% | 5987 | 1361 | 950 |
| 2 Politics and values 2011 | 159 | 6-12-2010 to 29-12-2010 | 3-1-2011 to 30-1-2011 | 73.60% | 5287 | 1595 | 950 |
| 3 Health 2009 | 249 | 2-11-2009 to 25-11-2009 | 7-12-2009 to 30-12-2009 | 66.70% | 6070 | 1558 | 950 |
| 4 Health 2011 | 249 | 7-11-2011 to 30-11-2011 | 5-12-2011 to 26-12-2011 | 77.60% | 5044 | 1690 | 950 |
| 5 Personality 2009 | 183 | 4-5-2009 to 27-5-2009 | 1-6-2009 to 30-6-2009 | 69.90% | 5614 | 1371 | 950 |
| 6 Personality 2011 | 183 | 2-5-2011 to 29-5-2011 | 6-6-2011 to 29-6-2011 | 76.30% | 5198 | 1653 | 950 |
| 7 Trauma 2012 | 82 | 1-4-2012 to 30-4-2012 |  | 78.40% | 5879 | see above | 950 |

^1^ PTE: Potentially traumatic events.

^2^ Selection of respondents confronted with one or more potential traumatic events in 2 years before survey on trauma 2012 (number of respondents participating in survey on trauma and one previous survey is equal to number in row of that specific survey).

^3^ N List wise: Number of respondents participating in all 7 surveys in table S1
