# Supplementary material for: The Burden of Research on Trauma for Respondents: A Prospective and Comparative Study on Respondents Evaluations and Predictors
Source: PLoS One. 2013 Oct 21;8(10):e77266. doi: 10.1371/journal.pone.0077266 (PMC3804544; doi:10.1371/journal.pone.0077266)
Supplement: Appendix S2 — (DOCX) [file pone.0077266.s002.docx]

|  | **N** | **Enjoy answering** | | | | | **Questionnaire get thinking** | | | | | **Interesting** | | | | | **Difficult to answer** | | | | | **Questions sufficiently** | | | | |
| --- | --- | --- | --- | --- | --- | --- | --- | --- | --- | --- | --- | --- | --- | --- | --- | --- | --- | --- | --- | --- | --- | --- | --- | --- | --- | --- |
|  | **Total** | **questions** | | | | | **about things** | | | | | **subject** | | | | | **the questions** | | | | | **clear** | | | | |
|  |  |  |  |  |  | **Cohen** | |  |  |  | **Cohen** | |  |  |  | **Cohen** | |  |  |  | **Cohen** | |  |  |  | **Cohen** |
| **Research topic** |  | **M** | **SD** | **T** | **p** | **D** | **M** | **SD** | **T** | **p** | **D** | **M** | **SD** | **T** | **p** | **D** | **M** | **SD** | **T** | **p** | **D** | **M** | **SD** | **T** | **p** | **D** |
| Politics&values 2009 | 289 | **3.68** | **0.90** | **5.66** | **<.001** | **0.33** | 2.87 | 1.05 | -5.97 | <.001 | **-0.35** | **3.69** | **0.96** | **0.93** | **ns.** |  | 2.18 | 1.17 | -0.79 | ns. |  | **4.10** | **0.99** | **-0.37** | **ns.** |  |
| Trauma 2012 | 289 | **3.27** | **1.10** |  |  |  | 3.32 | 1.27 |  |  |  | **3.62** | **1.08** |  |  |  | 2.26 | 1.37 |  |  |  | **4.13** | **1.04** |  |  |  |
| Politics&values 2011 | 499 | **3.82** | **0.99** | **7.94** | **<.001** | **0.36** | 3.21 | 1.08 | -4.30 | <.001 | **-0.19** | **3.81** | **0.96** | **2.50** | **0.01** | **0.11** | 2.27 | 1.33 | -2.02 | 0.04 | **-0.09** | **4.14** | **0.94** | **-0.15** | **ns.** |  |
| Trauma 2012 | 499 | **3.41** | **1.20** |  |  |  | 3.44 | 1.22 |  |  |  | **3.69** | **1.09** |  |  |  | 2.40 | 1.42 |  |  |  | **4.15** | **1.01** |  |  |  |
| Health 2009 | 471 | **3.64** | **1.03** | **4.73** | **<.001** | **0.22** | 2.54 | 1.14 | -13.82 | <.001 | **-0.64** | **3.46** | **1.06** | **-4.47** | **<.001** | **-0.21** | 1.77 | 1.04 | -8.78 | <.001 | **-0.40** | **4.07** | **1.11** | **-1.20** | **ns.** |  |
| Trauma 2012 | 471 | **3.36** | **1.15** |  |  |  | 3.40 | 1.21 |  |  |  | **3.70** | **1.04** |  |  |  | 2.37 | 1.40 |  |  |  | **4.15** | **1.01** |  |  |  |
| Health 2011 | 591 | **3.74** | **0.98** | **7.45** | **<.001** | **0.31** | 2.96 | 1.17 | -9.23 | <.001 | **-0.38** | **3.63** | **0.98** | **-0.23** | **ns.** |  | 1.76 | 1.06 | -10.00 | <.001 | **-0.41** | **4.22** | **0.99** | **2.03** | **0.04** | **0.08** |
| Trauma 2012 | 591 | **3.38** | **1.16** |  |  |  | 3.41 | 1.21 |  |  |  | **3.64** | **1.08** |  |  |  | 2.35 | 1.41 |  |  |  | **4.13** | **1.01** |  |  |  |
| Personality 2009 | 297 | **3.63** | **1.00** | **5.35** | **<.001** | **0.31** | 3.21 | 1.10 | -1.83 | <.001 | **-0.11** | **3.75** | **0.99** | **1.51** | **ns.** |  | 2.82 | 1.28 | 4.97 | <.001 | **0.29** | **3.95** | **0.96** | **-2.72** | **0.01** | **-0.16** |
| Trauma 2012 | 297 | **3.25** | **1.13** |  |  |  | 3.34 | 1.24 |  |  |  | **3.64** | **1.07** |  |  |  | 2.34 | 1.43 |  |  |  | **4.15** | **1.06** |  |  |  |
| Personality 2011 | 554 | **3.65** | **1.03** | **6.49** | **<.001** | **0.28** | 3.29 | 1.10 | -2.41 | 0.02 | **-0.10** | **3.75** | **1.00** | **2.72** | **0.01** | **0.12** | 2.44 | 1.27 | 0.40 | ns. |  | **4.00** | **0.98** | **-1.91** | **0.06** | **-0.08** |
| Trauma 2012 | 554 | **3.32** | **1.18** |  |  |  | 3.40 | 1.20 |  |  |  | **3.62** | **1.07** |  |  |  | 2.41 | 1.40 |  |  |  | **4.09** | **1.02** |  |  |  |

Appendix S2. Pair-wise comparisons of burden research on politics and values, personality and health, with burden of research on trauma among respondents not participating at all surveys.

p is p-value paired t-test, 2-tailed.

ns. = not significant.
