# Supplementary material for: The Burden of Research on Trauma for Respondents: A Prospective and Comparative Study on Respondents Evaluations and Predictors
Source: PLoS One. 2013 Oct 21;8(10):e77266. doi: 10.1371/journal.pone.0077266 (PMC3804544; doi:10.1371/journal.pone.0077266)
Supplement: Appendix S3 — (DOCX) [file pone.0077266.s003.docx]

Appendix S3. Full models multiple regression analyses predicting burden of research on trauma, with burden of surveys in

2009 as predictors (Step 6, N^max^=950).

|  | | | | | | | | | | | | | | | | | | | | |
| --- | --- | --- | --- | --- | --- | --- | --- | --- | --- | --- | --- | --- | --- | --- | --- | --- | --- | --- | --- | --- |
|  | **Enjoy answering** | | | | **Questionnaire get** | | | | **Interesting** | | | | **Difficult to answer** | | | | **Questions** | | | |
|  | **questions** | | | | **thinking about things** | | | | **subject** | | | | **the questions** | | | | **sufficiently clear** | | | |
|  | **B** | **SE** | **β** | **p** | **B** | **SE** | **β** | p | **B** | **SE** | **β** | **p** | **B** | **SE** | **β** | p | **B** | **SE** | **β** | **p** |
| PSS | **0.00** | **0.00** | **0.02** | **ns.** | 0.01 | 0.00 | 0.15 | <.001 | **0.01** | **0.00** | **0.16** | **<.001** | 0.01 | 0.00 | 0.12 | 0.00 | **0.00** | **0.00** | **0.07** | **ns.** |
| CSE | **0.03** | **0.01** | **0.21** | **<.001** | -0.01 | 0.01 | -0.06 | ns. | **0.01** | **0.01** | **0.08** | **.028** | -0.03 | 0.01 | -0.16 | <.001 | **0.02** | **0.01** | **0.16** | **<.001** |
| Education | **-0.05** | **0.03** | **-0.06** | **.066** | 0.00 | 0.03 | 0.00 | ns. | **-0.02** | **0.03** | **-0.03** | **ns.** | -0.01 | 0.03 | -0.01 | ns. | **-0.04** | **0.02** | **-0.06** | **ns.** |
| Age | **0.00** | **0.00** | **0.00** | **ns.** | 0.00 | 0.00 | -0.04 | ns. | **0.00** | **0.00** | **0.00** | **ns.** | 0.01 | 0.00 | 0.07 | .024 | **0.00** | **0.00** | **-0.02** | **ns.** |
| Income | **-0.02** | **0.02** | **-0.03** | **ns.** | -0.02 | 0.02 | -0.04 | ns. | **-0.03** | **0.02** | **-0.06** | **ns.** | 0.00 | 0.03 | 0.00 | ns. | **-0.01** | **0.02** | **-0.03** | **ns.** |
| Gender | **-0.02** | **0.07** | **-0.01** | **ns.** | -0.08 | 0.08 | -0.03 | ns. | **0.01** | **0.07** | **0.01** | **ns.** | -0.17 | 0.10 | -0.06 | ns. | **0.05** | **0.07** | **0.02** | **ns.** |
| Extraversion | **0.00** | **0.01** | **0.00** | **ns.** | -0.01 | 0.01 | -0.04 | ns. | **0.00** | **0.01** | **0.02** | **ns.** | 0.00 | 0.01 | -0.01 | ns. | **0.00** | **0.01** | **-0.01** | **ns.** |
| Agreeableness | **0.00** | **0.01** | **0.01** | **ns.** | 0.02 | 0.01 | 0.10 | .003 | **0.02** | **0.01** | **0.07** | **.047** | 0.01 | 0.01 | 0.03 | ns. | **0.02** | **0.01** | **0.12** | **.001** |
| Conscientiousness | **0.00** | **0.01** | **-0.01** | **ns.** | 0.01 | 0.01 | 0.04 | ns. | **0.00** | **0.01** | **0.00** | **ns.** | 0.00 | 0.01 | 0.01 | ns. | **0.01** | **0.01** | **0.07** | **.045** |
| Neuroticism | **0.01** | **0.01** | **0.05** | **ns.** | 0.00 | 0.01 | -0.01 | ns. | **0.00** | **0.01** | **0.02** | **ns.** | -0.01 | 0.01 | -0.07 | 0.04 | **0.01** | **0.01** | **0.04** | **ns.** |
| Openness | **0.00** | **0.01** | **0.00** | **ns.** | 0.01 | 0.01 | 0.03 | ns. | **0.01** | **0.01** | **0.03** | **ns.** | 0.00 | 0.01 | 0.01 | ns. | **0.01** | **0.01** | **0.03** | **ns.** |
| Politics 2009 | **0.17** | **0.04** | **0.15** | **<.001** | 0.17 | 0.04 | 0.15 | <.001 | **0.13** | **0.04** | **0.12** | **<.001** | 0.17 | 0.04 | 0.14 | <.001 | **0.00** | **0.03** | **0.00** | **ns.** |
| Health 2009 | **0.28** | **0.04** | **0.25** | **<.001** | 0.25 | 0.04 | 0.22 | <.001 | **0.17** | **0.04** | **0.16** | **<.001** | 0.14 | 0.05 | 0.10 | .002 | **0.16** | **0.03** | **0.16** | **<.001** |
| Personality 2009 | **0.21** | **0.04** | **0.18** | **<.001** | 0.27 | 0.04 | 0.24 | <.001 | **0.25** | **0.04** | **0.23** | **<.001** | 0.28 | 0.04 | 0.26 | <.001 | **0.18** | **0.03** | **0.18** | **<.001** |
| Type event | **-0.05** | **0.07** | **-0.02** | **ns.** | 0.02 | 0.07 | 0.01 | ns. | **-0.06** | **0.07** | **-0.03** | **ns.** | 0.01 | 0.09 | 0.00 | ns. | **0.00** | **0.06** | **0.00** | **ns.** |
| Time | **-0.04** | **0.06** | **-0.02** | **ns.** | 0.01 | 0.07 | 0.01 | ns. | **0.00** | **0.06** | **0.00** | **ns.** | 0.07 | 0.09 | 0.02 | ns. | **-0.05** | **0.06** | **-0.02** | **ns.** |

PSS =posttraumatic stress symptoms.

CSE= coping self-efficacy.

ns. = not significant.
